# Assessing Environment Resistance of the Korean Wild Boar ASFV Isolates “*ASFV/Yeoncheon/2019*”

**DOI:** 10.1155/tbed/4032319

**Published:** 2025-06-03

**Authors:** Hyunji Gu, Gun-Hee Lee, Yongkwan Kim, Dae-Min Kim, Eun-Gyeong Lee, Sang-Hyun Lee, Ji-Soo Kim, Garam Kim, So-Jeong Kim, Wonjun Kim, Youngsik Kim, Jae-Ku Oem, Ho-Seong Cho, Weonhwa Jheong, Dongseob Tark

**Affiliations:** ^1^Laboratory for Infectious Disease Prevention, Korea Zoonosis Research Institute, Jeonbuk National University, Iksan 54531, Republic of Korea; ^2^Wildlife Disease Response Team, National Institute of Wildlife Disease Control and Prevention, Gwangju 62407, Republic of Korea; ^3^College of Veterinary Medicine, Jeonbuk National University, Iksan 54596, Republic of Korea

## Abstract

African swine fever (ASF) is a disease characterized by high mortality and severe hemorrhagic damage in swine breeds, leading to significant economic losses for domestic pig farm operations. Although the introduction route of ASF in South Korea remains unclear, the primary transmission is believed to be through contamination of environmental fomites by migrating ASF-positive wild boar. Therefore, understanding the persistence of African swine fever virus (ASFV) under various environmental conditions in South Korea is crucial. In this study, we assessed the stability and infectivity of the *ASFV/Yeoncheon/2019* isolate under different experimental conditions, including variations in temperature and environmental matrices. Our results indicate that most infectious samples, except for excretions, maintained ASFV infectivity for 36–48 weeks, particularly at low temperatures. In contrast, excretion samples, such as feces and urine rapidly inactivate ASFV infectivity within 15 days, even when stored at low temperatures. Environmental matrices spiked with infectious blood samples showed rapid decrease in hemadsorption (HAD) titers within 9–15 days. However, the ASFV infectivity remained in most samples, which were previously deemed HAD negative. These findings suggest the need for caution when assessing virus inactivation and provide insight into the environmental persistence of ASFV in various matrices.

## 1. Introduction

African swine fever (ASF) is a devastating disease affecting domestic pigs and wild boar, leading to significant economic losses in livestock and posing a threat to wildlife due to the absence of a suitable vaccine. The causative agent, African swine fever virus (ASFV), is the sole member of the family Asfarviridae [1]. Infected hosts exhibit clinical signs, such as generalized hemorrhagic fever, diarrhea, and vomiting, with lethality rates approaching 100% [2]. ASFV transmission can occur both directly and indirectly. Direct transmission involves contact with ASFV-infected animals, including wild boar, or their infectious excretions and secretions. Indirect transmission occurs when healthy animals consume infected pork products or come into contact with contaminated fomites [3, 4]. Since its initial description in Africa, ASF has broadly spread across Europe, Russia, and several Asian countries, including China, Vietnam, Indonesia, North Korea, and South Korea [5, 6]. Following the emergence of ASFV in China, Korean authorities implemented stringent surveillance measures, such as restricting the import of pig products from affected regions, to prevent the introduction of ASFV. Despite these efforts, the first domestic case of ASFV was reported in 2019 at a pig farm in Paju city, Gyeonggi Province, near the border between South Korea and North Korea [7, 8]. Additionally, over 2600 cases of ASF-positive wild boar carcasses were reported in the region during the same period, with the disease spreading eastward and southward [5]. The short-distance spread of ASFV is attributed to the movement of ASF-positive wild boar, while long-distance spread may be facilitated by human activities involving contaminated soil and water [9]. The environmental resistance of ASFV is a critical factor in its spread. However, the survival of ASFV under the diverse environmental conditions of South Korea, which experiences four distinct seasons, is not fully understood. Several studies have investigated the stability and infectivity of ASFV under various experimental conditions [10, 11], examining its persistence in blood [10], excretions [11, 12], and different organs [13, 14]. Factors, such as virion structure, temperature, humidity, pH, and fomite surface material significantly impact virus stability in the environment [15, 16]. Given the variability of these factors across different regions, it is essential to establish the specific conditions required for ASFV inactivation in each country. In this study, we evaluated the stability of the *ASFV/Yeoncheon/2019* isolate in muscle, organs, blood, and excretions of domestic pigs under various experimental conditions, including different temperatures and environmental fomites, such as water and soil. Additionally, we assessed the ASFV activity in end point samples through further experiments. This study aims to determine the biosafety measures required in the field and to elucidate the epidemiological transmission of ASF in South Korea.

## 2. Materials and Methods

### 2.1. Cells and Virus

The pulmonary alveolar macrophages (PAMs) were collected by lung lavage from a 6-week-old female specific pathogen-free (SPF) White Yucatan pig (Optipharm, South Korea), as described [16]. The pulmonary alveolar macrophage (PAM) cells were cultured in RPMI 1640 (Thermo Fisher Scientific, USA) supplemented with 10% fetal bovine serum (FBS, Thermo Fisher Scientific, USA), 100 U/mL penicillin, and 100 µg/mL streptomycin (Thermo Fisher Scientific, USA). The *ASFV/Yeoncheon/2019* isolate (South Korea epidemic strain, genotype II) was obtained from the National Institute of Wildlife Disease Control and Prevention, South Korea. This isolate was maintained and propagated in PAM cell cultures. All in vitro ASFV experiments were performed in the biosafety level 3 (BSL-3) laboratory with personal protection equipment following the biosafety manual issued by the Korea Zoonosis Research Institute of Jeonbuk National University.

### 2.2. Analysis of ASFV Titration and Quantitative PCR (qPCR)

Following the manufacturer's guide, ASFV genomic DNA was purified by DNeasy Blood & Tissue kit (Qiagen, Netherlands). Purified DNA was quantified by the nanodrop (Thermo Fisher Science, USA) and stored at 4°C. qPCR was performed using a VetMAX ASFV Detection kit (Thermo Fisher Science, USA) for 2 min at 50°C, 10 min at 95°C, and then 45 cycles for 15 s at 95°C, and 1 min at 60°C following the manufacturer's P72 primer set. ASFV genome copies were calculated as *Ct* values from the standard curve. ASFV titration was calculated via hemadsorption (HAD) assay using supernatants of ASFV-infected PAM cells. Briefly, prepare diluents of 1 × 10^−8^ to 1 × 10^−1^ through 10-fold serial dilution of non-titrated ASFV using serum-free RPMI 1640 medium. PAM (3 × 10^4^ cells/well) cells were inoculated with diluents for three replicates and sequentially added fresh 1% swine erythrocytes isolated from swine blood through centrifugation. At 4–6 days postinfection (dpi), the formation of HAD in ASFV-infected cells was monitored, and HAD_50_ of ASFV was calculated by the Reed and Muench [17] method.

### 2.3. Animal Experiments

Three 6-week-old female White Yucatan pigs purchased from Optipharm Korea (Optipharm. Co. Ltd., South Korea) were inoculated with 1 × 10^4^ HAD_50_/mL *ASFV/Yeoncheon/2019* via the intramuscular (IM) injection. Each day after injection, ASFV shedding was analyzed in the nasal swab, rectal swab, and feces. Furthermore, pigs were analyzed for viremia and monitored for changes in body temperature and clinical signs until euthanized or died. Clinical scoring of each criterion was scored as described [18]. The scores for each criterion were summed, and a higher score indicated more severity. At 8 dpi, pigs infected with ASFV were necropsied to assess pathological lesions in various organs, including the tonsils, spleen, liver, heart, kidneys, lungs, and several lymph nodes. ASFV viremia was analyzed by qPCR from blood samples collected every 2–3 days after infection. Furthermore, the ASFV shedding was determined in rectal and nasal swabs using a clinical virus transport medium with an NS-1 swab applicator (Noble Biosciences Inc., South Korea) during the ASFV infection experiment. For the experiment, the muscle, blood, urine, feces, and spleen were collected from the hip, heart, bladder, and large intestine, respectively, during necropsy. All in vivo animal experiments using ASFV were conducted in animal biosafety level 3 (ABSL-3) compliance with the Animal Welfare Act. Animal experiments were approved by the institutional animal committee of the Jeonbuk National University (IACUC, JBNU 2022-082) and performed according to the guidelines of the institutional biosafety committee (IBC, JBNU 2022-03-001).

### 2.4. Design of Samples and Storage

At 8 dpi, the experimental contagious samples, muscle, spleen, blood, urine, and feces were obtained by necropsy. The spleen and muscle samples were cut into 1.5 and 3 g pieces. Feces obtained from large intestine were aliquoted into 1.5 g, while blood and urine obtained from the heart and bladder were aliquoted into 1 and 2 mL and placed into 50 mL tube (SPL Life Sciences, South Korea). These intact samples were stored in a refrigerator (GMS Co., Ltd, South Korea and JS Research Inc., South Korea) at three different temperatures (4°C, 13°C, and 22°C) that were determined using averages of four seasons from the South Korea Meteorological Administration. To mimic environmental conditions, samples were mixed and stored in three matrices: water and two different soils (D-soil and U-soil) at three different temperatures. An aliquot of each 9 mL water and 9 g soil was placed into a 50 mL tube and spiked with 1 mL of blood obtained from the ASFV-infected pig, respectively. All 50 mL tubes were sealed by parafilm (Amcor, Switzerland) to maintain humidity and analyze the ASFV activity during storage. The environmental matrices were collected from mountains and the river near the domestic pig farm where ASF occurred in Dongducheon (D-soil) and ASF-free Uijeongbu (U-soil and water). The composition of two different soils was analyzed at the safety accuracy process lab (South Korea), and water was analyzed at the clear water analysis research (South Korea).

### 2.5. ASFV Isolation From Samples

The supernatants of muscle and spleen samples were obtained through homogenization using a Precellys lysing kit (Bertin Technologies, France) with a serum-free RPMI 1640 medium. For qPCR, the supernatants were treated with 600 mAU/mL proteinase K (Qiagen, Netherlands) and subjected to ASFV genome extraction followed by DNeasy Blood and Tissue kit. In addition, the supernatants were 10-fold serial diluted with serum-free medium and ASFV infectivity was analyzed by HAD assay. The blood and urine samples were directly filtered with 0.45 µm filter (Merck Millipore, USA) and treated with proteinase K for ASFV genome extraction or 10-fold serial diluted with serum-free medium for HAD assay. The supernatant of the feces sample was obtained by pooling with serum-free medium and vortexing. The supernatant was filtered with a 0.45 µm filter and treated with proteinase K for ASFV genome extraction or 10-fold serial diluted with serum-free medium for HAD assay. The 1 g soil sample was placed into a new 50 mL tube and vortex after adding serum-free medium. The sample was incubated at room temperature (RT) for 20 min and the supernatant was filtered with 0.45 µm filter after pooling through freeze-thawing. The water sample was directly filtered with 0.45 µm filter and analyzed by qPCR and HAD assay.

### 2.6. Swine Bioassays

Swine bioassays were performed to detect infectious ASFV in the end point samples that had detected the ASFV genome on qPCR but not detected infectivity on HAD assay. Briefly, ASFV isolated from the end point samples were inoculated into PAM cells with 1% swine erythrocytes. At 96 h postinoculation, HAD formation in ASFV-inoculated cells was monitored by microscope (Leica Microsystems, Germany) and the supernatant was collected by freeze-thawing. Two 6-week-old female White Yucatan pigs were infected with the supernatant via the IM injection, while the negative control was inoculated with a serum-free medium. Every day after infection, pigs were monitored for changes in clinical signs. The ASFV viremia was analyzed in pig blood when collected at 6 dpi. Furthermore, the ASFV shedding was determined by nasal and rectal swabs when collected every 3 dpi. At 12 dpi, all pigs were euthanized and necropsied to assess clinical lesions in several organs.

### 2.7. Statistical Analysis

Three independent experiments represented data as the mean ± standard deviations (SDs). Statistical analysis was performed using GraphPad Prism 8.0.1 software.

## 3. Results

### 3.1. Evaluation of the Virulence of ASFV/Yeoncheon/2019 Isolate in Domestic Pig

**Figure 1:**
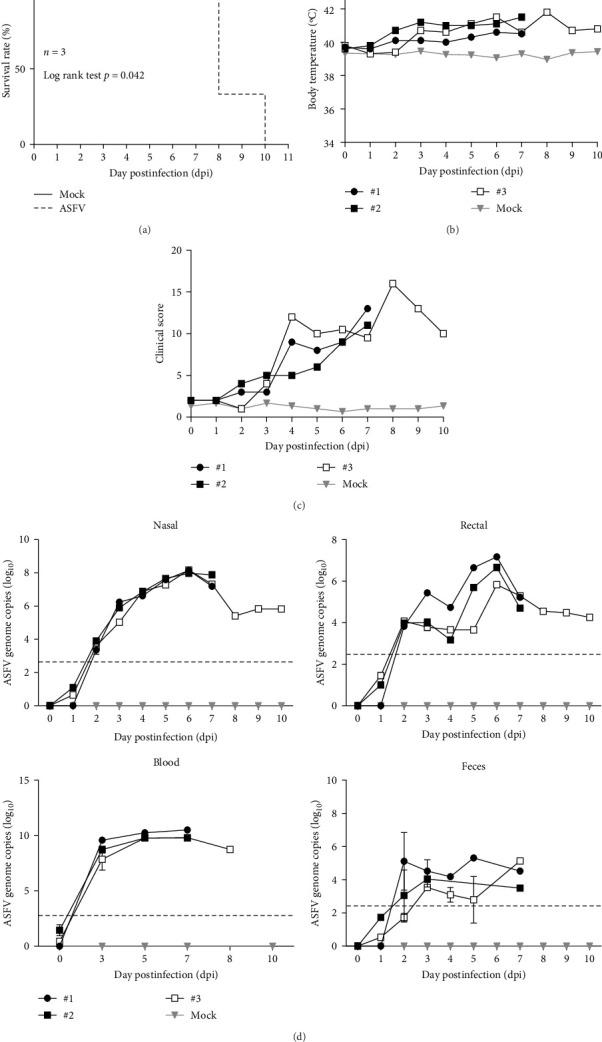
Analysis of pathogenicity in experimental pigs infected with ASFV/Yeoncheon/2019 isolate. Three 6-week-old female White Yucatan Pigs were inoculated with 1 x 10^4^ HAD_50_/mL ASFV/Yeoncheon/2019 isolate via the IM injection. (a) Survival rate, (b) body temperature, (c) clinical scoring were monitored daily in White Yucatan pigs after inoculation, and (d) ASFV genome copies in the feces, blood, nasal, and rectal swabs were quantified by qPCR at indicated times after injection. The limited detection for ASFV genome copies was represented by a dotted line at 2.4 log_10_ copies. Representative data were analyzed using GraphPad Prism 8.0.1 software and presented as the mean ± standard error of the mean (SEM) value.

We inoculated three 6-week-old female White Yucatan pigs with 1 x 10^4^ HAD_50/_mL *ASFV/Yeoncheon/2019* via IM injection (*n* = 3). Clinical signs were monitored for 10 dpi. The infected pigs exhibited high lethality, two succumbed at 8 dpi and one at 10 dpi. The infected pigs also showed a significant increase in body temperature, exceeding 40°C, compared to the mock control group (Figure 1a,b). Additionally, the infected pigs displayed high clinical scores, consistently exhibiting signs, such as fever, cyanosis, and anorexia throughout the infection period (Figure 1c). The necropsy revealed severe hemorrhagic damage in multiple organs, including the spleen, kidneys, and various lymph nodes (Supporting Information Figure S1). We monitored ASFV shedding in nasal swabs, rectal swabs, and feces daily. ASFV genome copies in the blood remained high (average 10 log_10_) and in nasal and rectal swabs peaked at 6 dpi (8 log_10_ and 7 log_10_, respectively), while fecal samples showed lower ASFV genome copies with significant individual variation (Figure 1d). Furthermore, similar ASFV genome copies were detected in various tissues and lymph nodes obtained through necropsy, depending on the individual pigs (Supporting Information Figure S2). These results indicate that the *ASFV/Yeoncheon/2019* isolate exhibits similar clinical signs, virus shedding, and viral replication in tissues compared to other highly virulent ASFV strains.

### 3.2. Design and Sample Collection for the Studies in the Environmental Experiment

**Figure 2:**
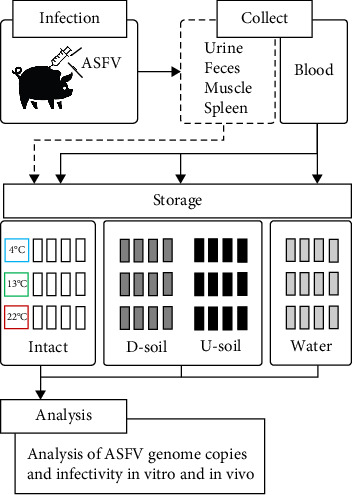
Schematic of experimental design. Samples obtained from ASFV-infected pigs were stored alone (Intact) or on environmental matrices, such as D-soil, U-soil, or water. Briefly, samples were collected from ASFV-infected pigs through necropsy. Infectious excretions, muscle, and spleen (dotted line) were stored intact at three different temperatures. The infectious blood (solid line) was stored intact or spiked in D-soil, U-soil, and water, and then stored at three different temperatures. The ASFV genome copies and infectivity were analyzed by qPCR and HAD assay throughout the experiment.

**Figure 3:**
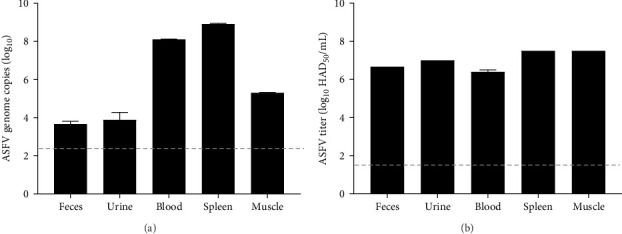
Evaluation of infectious ASFV in standard necropsy samples. (a) Quantification of ASFV genome copies in standard samples was performed using qPCR. (b) Infectious ASFV titers in standard samples were assessed via HAD assay. Data are presented as mean ± standard deviation (SD) from three independent experiments, analyzed using GraphPad Prism 8.0.1 software. The dotted line indicates the qPCR detection limit of 2.4 log_10_ copies and indicates the ASFV titer detection limit of 1.5 log_10_ HAD_50_/mL.

To study the environmental resistance of ASFV, stored samples were prepared according to the conditions in Figure 2 and as described in the materials and methods. Briefly, samples were collected from White Yucatan pigs infected with ASFV and environmental matrices from the mountains and the river near the domestic pig farm where ASF occurred, or from an ASF-free region (Supporting Information Figure S3). The contagious samples and environmental matrices were mixed and then stored at three different temperatures (Figure 2). First, we established the viral genome copies and titer of infectious ASFV in contagious samples obtained through necropsy at 8 dpi to serve as a standard for experiments. Low levels of ASFV genome copies were detected in excretions, with 3.6 log_10_ in feces and 3.8 log_10_ in urine samples. In contrast, high levels were detected at 8.1 log_10_ in blood and 8.9 log_10_ in the spleen, with moderate levels at 5.3 log_10_ in muscle samples (Figure 3a). Moreover, high levels of infectious ASFV have remained in all contagious samples up to 6 log_10_HAD_50_/mL (Figure 3b). Based on these results, all experimental samples were analyzed to the end point of ASFV stability and infectivity over storage times.

### 3.3. Persistence of ASFV in Intact Samples at Three Different Temperatures

**Figure 4:**
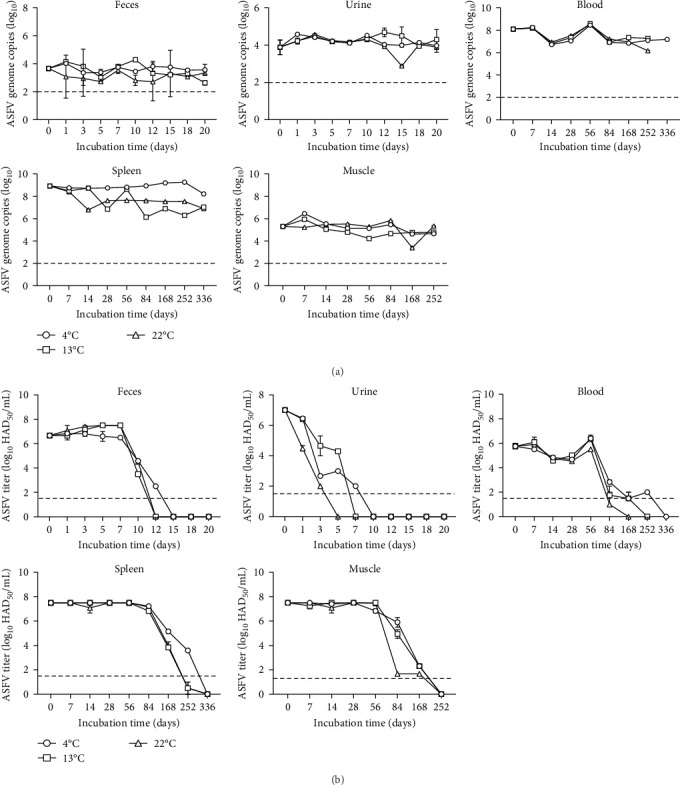
The stability and infectivity of ASFV were monitored during storage at three different temperatures. (a) The genome copies of ASFV in intact samples stored at three different temperatures were measured by qPCR at regular intervals. (b) The titration of infectious ASFV in intact samples stored at three different temperatures was performed using the HAD assay. Representative data show the mean and standard deviation (SD) from three independent experiments, analyzed using GraphPad Prism 8.0.1 software. The dotted line indicates the qPCR detection limit of 2 log_10_ copies and indicates the ASFV titer detection limit of 1.5 log_10_ HAD_50_/mL.

To investigate the stability of the ASFV genome and infectious ASFV titer in intact samples, we conducted analyses at regular intervals depending on the sample being stored. ASFV genome copies were detected in feces and urine for up to 20 days at three different temperatures. Additionally, the ASFV genome was confirmed for up to 252 days in blood and muscle, and up to 336 days in the spleen at three different temperatures (Figure 4a). Although the ASFV genome slightly decreased over time, but not significant. In contrast, infectious ASFV titers were reduced from day 7, with no infectious virus detected by day 15 at 4°C and day 12 at 13°C and 22°C in feces samples. In urine samples stored at 4°C and 13°C, infectious ASFV titers reduced from day 1, with no infectious virus detected on days 10 and 7, respectively. Infectious ASFV titers in urine samples at 22°C reduced rapidly immediately after storage, with no infectious virus detected by day 5. In blood samples, infectious ASFV titers markedly reduced from day 56 and were inactivated by days 336, 252, and 168 when stored at 4°C, 13°C, and 22°C, respectively. Inactivation of infectious ASFV was observed by day 336 in the spleen and day 252 in muscle samples stored at three different temperatures (Figure 4b). Taken together, our results indicate that the detection of the ASFV genome and infectivity are not correlated, and infectious ASFV shows different inactivation rates depending on the storage time and temperature of the intact samples.

### 3.4. Persistence of ASFV in Environmental Matrices at Three Different Temperatures

**Figure 5:**
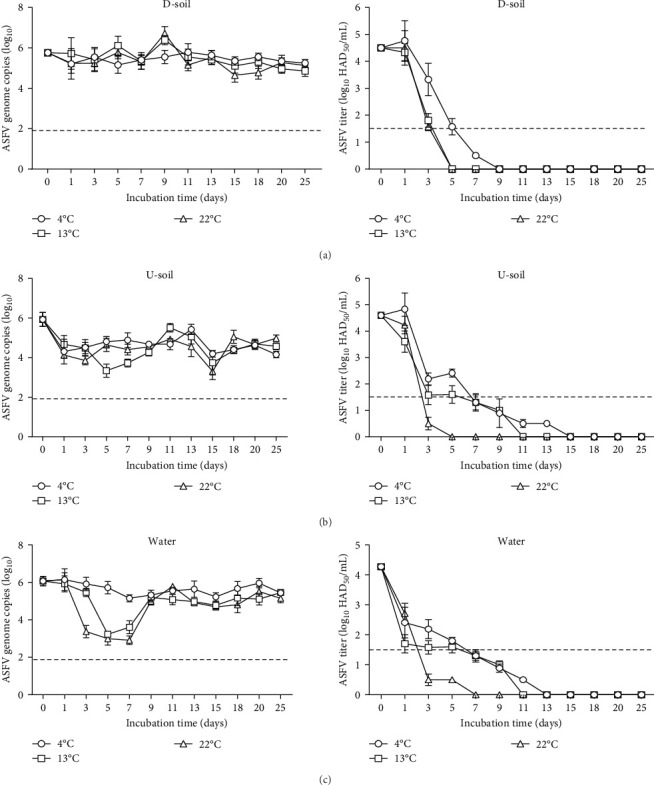
Establishment of infectious ASFV persistence in environmental matrices containing infectious blood stored at three different temperatures. The ASFV genome copies and titers were measured at regular intervals in (a) D-soil, (b) U-soil, and (c) water samples. The dotted line represents the qPCR detection limit of 1.9 log_10_ copies and indicates the ASFV titer detection limit of 1.5 log_10_ HAD_50_/mL. Representative data are presented as mean ± standard deviation (SD) from three independent experiments, analyzed using GraphPad Prism 8.0.1 software.

We analyzed the ASFV genome and infectious ASFV titer in environmental matrices containing contagious blood stored simultaneously with intact samples at three different temperatures. In D-soil (pH 4.5), ASFV titers of 4.5 log_10_ HAD_50_/mL were detected after spiking of infectious blood on day 0, but inactivation of ASFV was observed from 9 days in 4°C stored samples and from 5 days in 13°C and 22°C stored samples until the end of the experiment (Figure 5a). In U-soil (pH 5.8), a similar ASFV inactivation pattern to D-soil was observed, with inactivation occurring by day 15 at 4°C, day 11 at 13°C, and day 5 at 22°C (Figure 5b). In water (pH 7), low ASFV titers were detected by day 1 after spiking with infectious blood. Inactivation of ASFV was observed from day 13 at 4°C, day 11 at 13°C, and day 7 at 22°C (Figure 5c). However, the ASFV genome remained detectable in all environmental matrices, with no significant changes in ASFV genome copies observed during the experiment.

### 3.5. Evaluation of Inactivated ASFV Pathogenicity in Swine Bioassay

To evaluate the potential presence of infectious ASFV in all samples confirmed to have reached end points by HAD assay, including environmental samples, we determined ASFV infectivity using an ASFV susceptible cell-based passage. Therefore, we examined the infectiousness of ASFV in all environmental samples after the end point (Supporting Information Table S1). Briefly, ASFV isolated from the end point sample was inoculated into PAM cells. The supernatant was obtained from PAM inoculated with ASFV, and reinoculated into new PAM cells using the supernatant. After 5 days, the supernatants were collected from PAM cells and ASFV titers were measured by HAD assay to determine ASFV activity. Based on this analysis, we determined the final end point samples at which ASFV was inactivated. To test the presence of ASFV that was not detected in vitro, the final end point samples were inoculated intramuscularly into White Yucatan pigs to observe signs of ASFV infection. At 12 dpi, pigs were euthanized and necropsied to evaluate clinical lesions in several organs. In all end point samples, ASFV genome copies in viremia, nasal, and rectal swabs were relatively undetectable at 12 dpi compared to the positive control. Body temperature remained constant, and no lethality or clinical score changes were observed during the swine bioassay of all tested samples (Table 1). Additionally, clinical lesions in several organs caused by ASFV infection were not observed through necropsy (data not shown).

## 4. Discussion

Since the ASF introduction, ASFV has established self-sustaining and has a complex transmission cycle in target animal populations, such as wild boar and domestic pigs. The spread of ASF transmission in South Korea has been observed, mainly in pig farms, along the migration route of wild boars at a particular season of the year [19]. The Korean government's stringent biosafety measures effectively control the large-scale spread of ASFV infections in pig farms. However, ASFV is no longer classified as an exotic disease. Although the introduction of ASFV into South Korea has not yet been defined, the ecological persistence of ASFV is maintained by various factors, such as genetic evolution [20], contaminated fomites, and other biotic and abiotic factors [8]. The environmental matrices contaminated by wild boars infected with ASFV are recognized as one of the risk factors that could play a role in transmission [14, 21]. Evidence suggested that many ASFV-infected wild boars were found near the pig farms where ASF occurred (see map at https://mafra.go.kr/FMD-AI2/map/ASF/ASF_map.jsp).

Our study assessed infectious ASFV and genome stability in the blood, muscle, spleen, and excretions of experimentally ASFV-infected pigs stored at three different temperatures. Furthermore, we tested the potential risk for ASFV transmission in soil and water spiked with infectious blood stored at three different temperatures. The ASFV stability and infectivity in natural fields are influenced by numerous environmental variables, such as wind, humidity, rain, temperature, soil type, pH, and natural organic matter [13, 22, 23]. In our study, we had to choose temperature and matrices among the important environmental variables due to the limitations of the BSL3 experimental facility. The whole pig carcass infected with ASFV was unfeasible to store in laboratory conditions but only stored parts of them. The micro-conditions of ASFV-infected whole body decomposing in a natural environment [24] differ from those of body parts stored in the experimental setting. In addition, the qPCR detection limit varied depending on sample type. Results differed between immediately analyzed sample collected from pig and sample incubated over time under different conditions before analysis. Therefore, the results of this study should fully consider that the selective experimental conditions affected ASFV genome stability and infectivity.

Our results determine that the *ASFV/Yeoncheon/2019* isolate is stable, particularly long-term infectivity in all stored samples except the excretion samples at experimental conditions. Previously reported low temperatures contributed benefits for ASFV survival and environmental resistance [25, 26]. At 4°C, ASFV infectivity remains for at least 61 weeks when contained in the medium, and storing at 4°C preserves infectivity of viraemic blood for at least 75 weeks [27]. Another reported that ASFV infectivity in the blood remains for at least 18 months [28]. Our results are contradictory to previously published studies, we considered that the persistence period of ASFV infectivity at low temperatures may be linked to each experimental condition. Therefore, results observed that ASFV infectivity in the spleen, muscle, and blood had persisted longer at least 48, 36, and 48 weeks respectively under our experimental conditions, especially at low temperatures (Figure 4). Estimated ASFV survival in excretion has been reported that isolated infectious ASFV from urine for up to 5 days at 4°C and RT and from feces for up to 5 days at 4°C and 3 days stored at RT [11]. The presence of infectious ASFV in excretions stored at three different temperatures quickly decreased within 15 days, depending on temperature conditions (Figure 4). Our results showed that infectious ASFV titers in excretion samples were much higher than genome copies due to excretion often containing various substances, such as salts, complex polysaccharides, and metabolic byproducts that may interfere with the efficiency of PCR reaction [29, 30]. The long-term persistence of ASFV infectivity in low-temperature conditions may partly contribute to the spread of ASFV during South Korea's winter [19]. On the other hand, we observed the ASFV infectivity in end point excretion samples using ASFV-susceptible cells (data not shown). These findings indicate the limitations of the HAD assay for determining ASFV infectivity and the necessity to improve methods for infectious ASFV analysis [10]. Thus, we detected the ASFV infectivity through HAD assay and additional experiments, such as ASFV reproduction using ASFV susceptible cells. As a result, we determined that the infectious ASFV was completely inactivated later than each end point.

The soil and water comprise many components in nature and enable altered ecosystems by interaction with animals, microorganisms, temperatures, and location. They are important as fomites of local transmission through contact with carcasses of wild boars infected with ASFV [21]. We demonstrated that soil and water cause infectious ASFV to reach its end point faster than intact samples (Figure 5). The ASFV infectivity in soil and water was completely inactivated within 7–25 days, depending on the temperature, as confirmed by our additional experiments (Table 1). According to WOAH [22] and Homeland Security [31], ASFV can be inactivated below pH 3.9 and above pH 11.5 in serum-free medium. Previously reported re-isolated ASFV from the soil is reliably inactivated for a short 3-day incubation, independent of temperature conditions [6]. In addition, infectious ASFV could not recover from acidic forest soils (pH 4.1 and 3.2), even immediately after the application of infectious blood [32]. Our study used two soils described as D-soil, pH 4.5, and U-soil, pH 5.8 (Supporting Information Table S2). Infectious ASFV in contact with D-soil or U-soil completely lose their activity within 7–13 days at 13°C and 22°C and within 25 days at 4°C. However, the reduction ratio of infectious ASFV was reduced faster in D-soil than in U-soil. Although there are differences in composition, D-soil can be commonly found in South Korea (Average pH 4.7). The U-soil pH seems to be affected by the organic matter concentration, which is about 10% higher than D-soil [33]. Based on these results, we expected that infectious viral activity in natural soil would be reduced in a shorter period than our results due to various environmental factors. The ASFV survival in river water has not yet been exclusively studied. A recent publication [34] has described that ASFV *BA71V* maintains its stability and infectivity for more than 42 days in various river water samples when incubated at 4°C. In contrast, infectious ASFV was significantly reduced in the early period depending on storage temperatures in our experimental water samples (Figure 5). These differences are interpreted to occur after contact with water, and partial experimental conditions are also thought to affect the results.

The ASFV genome has been detectable in most experimental samples for longer than infectious ASFV. Although PCR is a standard and useful technique to confirm wide-range ASFV-positive, its relative viral survival is controversial, and caution should be exercised when assessing the ASFV infection risk. Therefore, we recommend sufficient cross-validation through HAD assay to determine ASFV infectivity. Furthermore, we suggest two additional experiments to confirm ASFV infectivity might be useful in detecting negative HAD results at least in samples of particular importance. Detecting ASFV inactivation in wild environmental conditions is important data underlying ASFV control and the national quarantine of the Korean government. Although inconsistent with several previous studies, our experiments mimicking wild environmental conditions suggest findings regarding the local-specificity of ASFV stability. Finally, we believe that future studies considering other factors will help to better understand the characteristics of ASFV in the domestic environment.

## 5. Conclusions

In this study, we investigated the stability of the *ASFV/Yeoncheon/2019* isolate under experimental conditions that mimic the South Korean environment. Our results indicate that viable ASFV was detected in muscle, spleen, and blood for several months, especially when stored at low temperatures, but only in excretions for up to 20 days. Additionally, we observed that contact between viable ASFV and environmental fomites, such as soil and water disrupted the persistence of ASFV infectivity, depending on storage temperatures. Therefore, we conclude that low temperatures in wild environments play a crucial role in sustaining ASFV infection over extended periods. Furthermore, assessing ASFV infectivity requires improved methods to detect the presence of infectious ASFV in various environments. Finally, our findings support the implementation of effective national quarantine measures through understanding the environmental resistance of ASFV.

## Figures and Tables

**Table 1:** Evaluation of end point environmental samples in swine bioassay.

Samples	Temperature (°C)	End point (days)	Body temperature (mean ± SD)	Clinical score	No. of survivor/total	Genome copies (viremia)	Genome copies (nasal swab)	Genome copies (rectal swab)
D-soil	4	25	39.12 ± 0.13	0	2/2	N.D	N.D	N.D
	13	7	39.8 ± 0.1	0	2/2	N.D	N.D	N.D
	22	7	40 ± 0.08	0	2/2	N.D	N.D	N.D

U-soil	4	25	38.98 ± 0.4	0	2/2	N.D	N.D	N.D
	13	13	39.84 ± 0.27	0	2/2	N.D	N.D	N.D
	22	7	39.26 ± 0.23	0	2/2	N.D	N.D	N.D

Water	4	25	39.64 ± 0.22	0	2/2	N.D	N.D	N.D
	13	25	39.36 ± 0.39	0	2/2	N.D	N.D	N.D
	22	9	39.12 ± 0.37	0	2/2	N.D	N.D	N.D

Negative control	—	—	39.32 ± 0.13	0	2/2	N.D	N.D	N.D

Positive control	—	—	40.5 ± 0.52	9.5	0/2	9.45 ± 0.71	6.5 ± 0.68	4.73 ± 0.48

## Data Availability

The data supporting the findings of this study are available from the corresponding author upon reasonable request.

## Supporting Information

Supporting Information 1 Figure S1: Determination of clinical lesions in multiple organs of White Yucatan pigs infected with ASFV/Yeoncheon/2019 isolate through necropsy. Severe hemorrhagic damage was observed in ASFV-infected pigs compared to mock. Figure S2: Measurement of ASFV genome copies in multiple tissues of White Yucatan pigs (#1, #2, and #3) infected with ASFV/Yeoncheon/2019 isolate. Each indicated tissue was obtained by necropsy. LN, Lymph node. The dotted line indicates the limit of detection for qPCR, 2.4 log10/copies. Figure S3: Spatial distribution of ASFV-positive farms (yellow circle) and environmental matrices collected places, Dongducheon – D-soil (red circle), Uijeongbu – U-soil (green circle), and water (blue circle).

Supporting Information 2 Table S1: ASFV infectivity from end point samples. Table S2: Soil and water analysis results.

## References

[1] Alonso C., Borca M., Dixon L. (2018). ICTV Virus Taxonomy Profile: Asfarviridae. *The Journal of General Virology*.

[2] Sánchez-Vizcaíno J. M., Mur L., Gomez-Villamandos J. C., Carrasco L. (2015). An Update on the Epidemiology and Pathology of African Swine Fever. *Journal of Comparative Pathology*.

[3] Mur L., Martinez-Lopez B., Sánchez-Vizcaíno J. M. (2012). Risk of African Swine Fever Introduction into the European Union Through Transport-Associated Routes: Returning Trucks and Waste From International Ships and Planes. *BMC Veterinary Research*.

[4] Guinat C., Gogin A., Blome S. (2016). Transmission Routes of African Swine Fever Virus to Domestic Pigs: Current Knowledge and Future Research Directions. *Veterinary Record*.

[5] Lim J. S., Andraud M., Kim E., Vergne T. (2023). Three Years of African Swine Fever in South Korea (2019-2021): A Scoping Review of Epidemiological Understanding. *Transboundary and Emerging Diseases*.

[6] Dixon L. K., Stahl K., Jori F., Vial L., Pfeiffer D. U. (2020). African Swine Fever Epidemiology and Control. *Annual Review of Animal Biosciences*.

[7] Kim H. J., Cho K. H., Lee S. K. (2020). Outbreak of African Swine Fever in South Korea, 2019. *Transboundary and Emerging Diseases*.

[8] Kim Y. J., Park B., Kang H. E. (2021). Control Measures to African Swine Fever Outbreak: Active Response in South Korea, Preparation for the Future, and Cooperation. *Journal of Veterinary Science*.

[9] Jo Y. S., Gortazar C. (2020). African Swine Fever in Wild Boar, South Korea, 2019. *Transboundary and Emerging Diseases*.

[10] Fischer M., Hühr J., Blome S., Conraths F. J., Probst C. (2020). Stability of African Swine Fever Virus in Carcasses of Domestic Pigs and Wild Boar Experimentally Infected With the ASFV “Estonia 2014, Isolate. *Viruses*.

[11] Davies K., Goatley L. C., Guinat C. (2017). Survival of African Swine Fever Virus in Excretions From Pigs Experimentally Infected With the Georgia 2007/1 Isolate. *Transboundary and Emerging Diseases*.

[12] de Carvalho Ferreira H. C., Weesendorp E., Quak S., Stegeman J. A., Loeffen W. L. A. (2014). Suitability of Faeces and Tissue Samples as a Basis for Non-Invasive Sampling for African Swine Fever in Wild Boar. *Veterinary Microbiology*.

[13] Mazur-Panasiuk N., Wozniakowski G. (2020). Natural Inactivation of African Swine Fever Virus in Tissues: Influence of Temperature and Environmental Conditions on Virus Survival. *Veterinary Microbiology*.

[14] Zani L., Masiulis M., Bušauskas P. (2020). African Swine Fever Virus Survival in Buried Wild Boar Carcasses. *Transboundary and Emerging Diseases*.

[15] French A. J., Longest A. K., Pan J. (2023). Environmental Stability of Enveloped Viruses Is Impacted by Initial Volume and Evaporation Kinetics of Droplets. *MBio*.

[16] Carrascosa A. L., Bustos M. J., de Leon P. (2011). Methods for Growing and Titrating African Swine Fever Virus: Field and Laboratory Samples. *Current Protocols in Cell Biology*.

[17] Reed L. J., Muench H. (1938). A Simple Method of Estimating Fifty per Cent Endpoints. *American Journal of Epidemiology*.

[18] King K., Chapman D., Argilaguet J. M. (2011). Protection of European Domestic Pigs From Virulent African Isolates of African Swine Fever Virus by Experimental Immunisation. *Vaccine*.

[19] Jo Y. S., Gortazar C. (2021). African Swine Fever in Wild Boar: Assessing Interventions in South Korea. *Transboundary and Emerging Diseases*.

[20] Chen S., Wang T., Luo R. (2024). Genetic Variations of African Swine Fever Virus: Major Challenges and Prospects. *Viruses*.

[21] Probst C., Globig A., Knoll B., Conraths F. J., Depner K. (2017). Behaviour of Free Ranging Wild Boar Towards Their Dead Fellows: Potential Implications for the Transmission of African Swine Fever. *Royal Society Open Science*.

[22] WOAH African Swine Fever. https://www.woah.org/app/uploads/2021/03/a-african-swine-fever-v2-0.pdf.

[23] Rogoll L., Güttner A. K., Schulz K. (2023). Seasonal Occurrence of African Swine Fever in Wild Boar and Domestic Pigs in EU Member States. *Viruses*.

[24] Benninger L. A., Carter D. O., Forbes S. L. (2008). The Biochemical Alteration of Soil beneath a Decomposing Carcass. *Forensic Science International*.

[25] Mazur-Panasiuk N., Zmudzki J., Wonzniakowski G. (2019). African Swine Fever Virus – Persistence in Different Environmental Conditions and the Possibility of Its Indirect Transmission. *Journal of Veterinary Research*.

[26] Sánchez-Vizcaíno J. M., Martínez-López B., Martínez-Avilés M. (2009). Scientific Review on African Swine Fever. *EFSA Journal*.

[27] Plowright W., Parker J. (1967). The Stability of African Swine Fever Virus With Particular Reference to Heat and pH Inactivation. *Archiv fur die gesamte Virusforschung*.

[28] EFSA Panel on Animal Health and Welfare (2010). EFSA Panel on Animal Health and Welfare, Scientific Opinion on African Swine Fever. *EFSA Journal*.

[29] Schrader C., Schielke A., Ellerbroek L., Johne R. (2012). PCR Inhibitors – Occurrence, Properties and Removal. *Journal of Applied Microbiology*.

[30] Jevtuševskaja J., Krõlov K., Tulp I., Langel Ü. (2017). The Effect of Main Urine Inhibitors on the Activity of Different DNA Polymerases in Loop-Mediated Isothermal Amplification. *Expert Review of Molecular Diagnostics*.

[31] Homeland Security Master Question List of African Swine Fever Virus (ASFV). https://www.dhs.gov/sites/default/files/2023-07/2023_06_07_st_asfv_mql.pdf.

[32] Carlson J., Fischer M., Zani L. (2020). Stability of African Swine Fever Virus in Soil and Options to Mitigate the Potential Transmission Risk. *Pathogens*.

[33] Korean Soil Information System (KSIS) Soil Chemical Properties. https://soil.rda.go.kr/eng/atlas/chemical.jsp.

[34] Loundras E. A., Netherton C. L., Flannery J., Bowes M. J., Dixon L., Batten C. (2023). The Effect of Temperature on the Stability of African Swine Fever Virus BA71V Isolate in Environmental Water Samples. *Pathogens*.

