# Supplementary figures and images for: Assessing Environment Resistance of the Korean Wild Boar ASFV Isolates “ASFV/Yeoncheon/2019”
Source: Transbound Emerg Dis. 2025 Jun 3;2025:4032319. doi: 10.1155/tbed/4032319 (PMC12151626; doi:10.1155/tbed/4032319)

Sup Figure 1

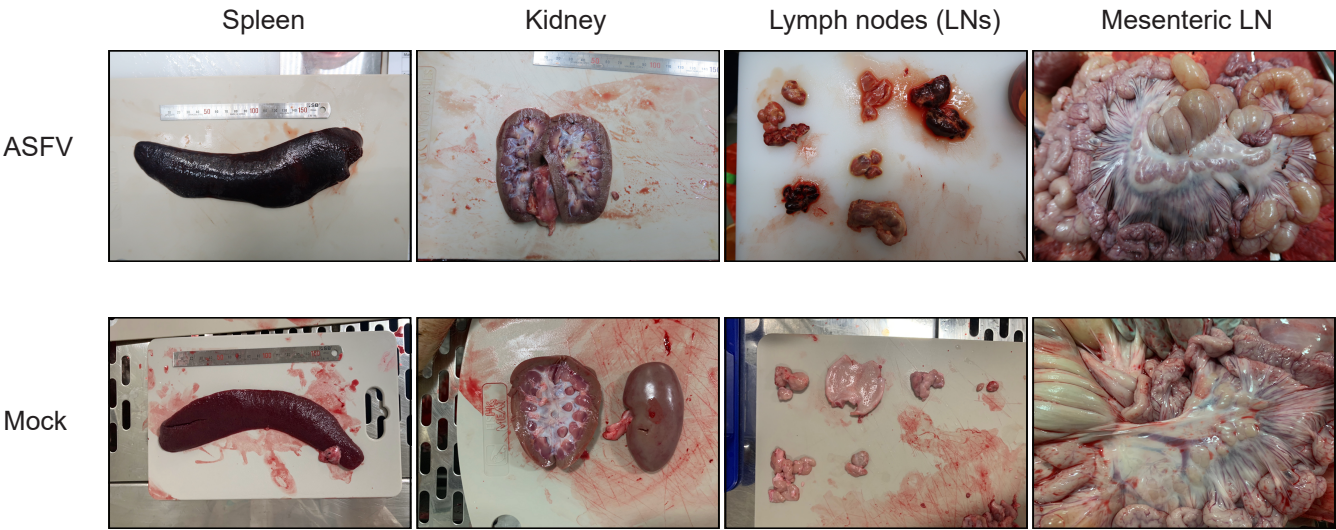

Sup Figure 2

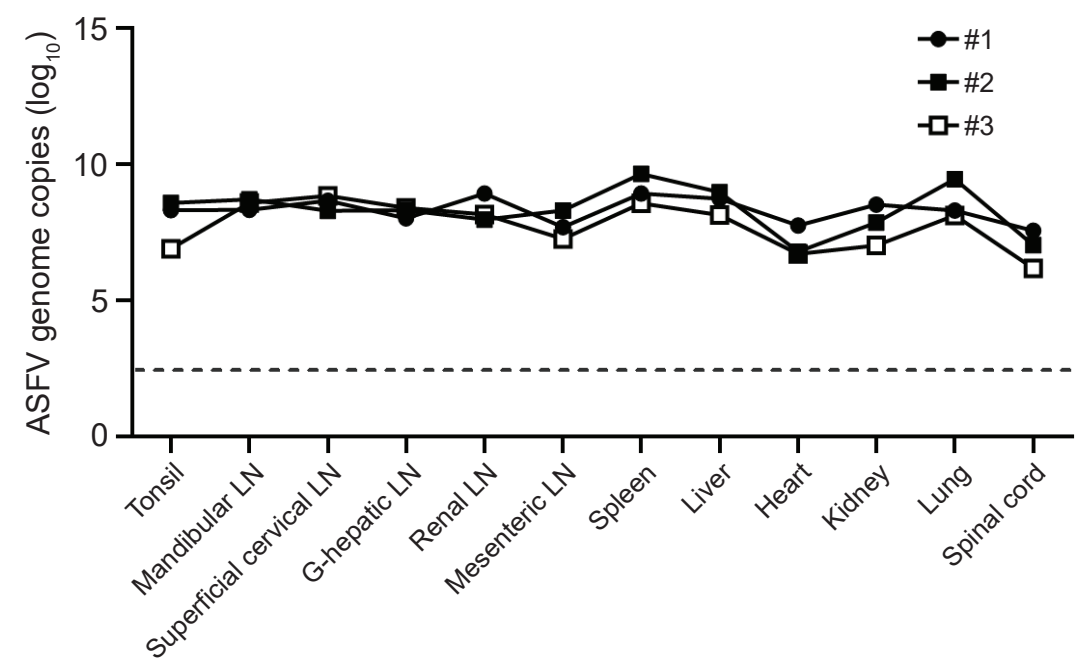

Sup Figure 3

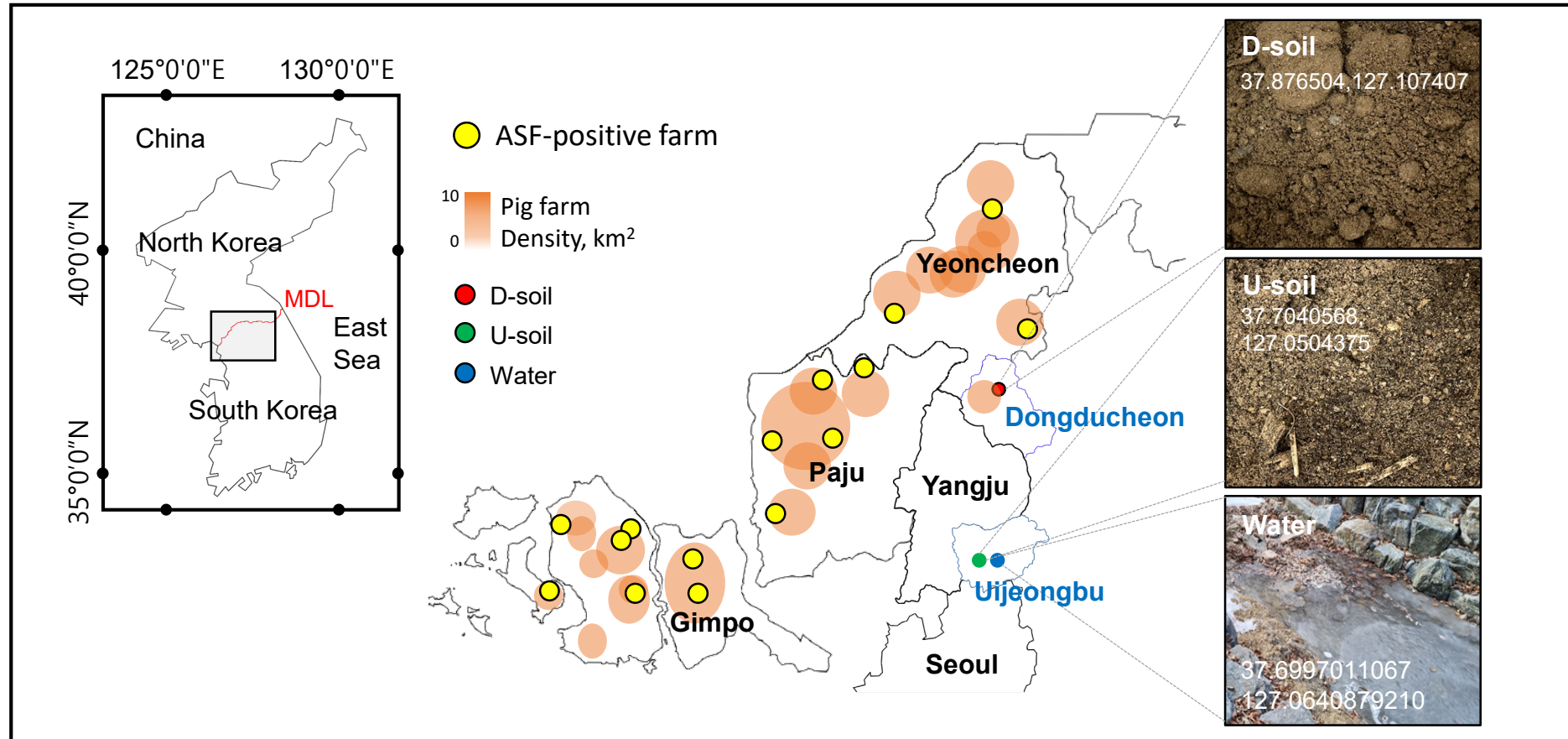

Supplement: Supporting Information 1 — Figure S1: Determination of clinical lesions in multiple organs of White Yucatan pigs infected with ASFV/Yeoncheon/2019 isolate through necropsy. Severe hemorrhagic damage was observed in ASFV-infected pigs compared to mock. Figure S2: Measurement of ASFV genome copies in multiple tissues of White Yucatan pigs (#1, #2, and #3) infected with ASFV/Yeoncheon/2019 isolate. Each indicated tissue was obtained by necropsy. LN, Lymph node. The dotted line indicates the limit of detection for qPCR, 2.4 log10/copies. Figure S3: Spatial distribution of ASFV-positive farms (yellow circle) and environmental matrices collected places, Dongducheon – D-soil (red circle), Uijeongbu – U-soil (green circle), and water (blue circle). [file 4032319.f1.pdf]
