# Supplementary material for: Assessing Environment Resistance of the Korean Wild Boar ASFV Isolates “ASFV/Yeoncheon/2019”
Source: Transbound Emerg Dis. 2025 Jun 3;2025:4032319. doi: 10.1155/tbed/4032319 (PMC12151626; doi:10.1155/tbed/4032319)
Supplement: Supporting Information 2 — Table S1: ASFV infectivity from end point samples. Table S2: Soil and water analysis results. [file 4032319.f2.pdf]

Supplementary Table 1: ASFV infectivity from endpoint samples

Temperature	4°C		13°C		22°C	
	Endpoint (days)	HA formation	Endpoint (days)	HA formation	Endpoint (days)	HA formation
D-soil	9	Detected	5	Detected	5	Detected
	11	Detected	7	N.D	7	N.D
	13	Detected				
	15	Detected				
	18	Detected				
	20	Detected				
	25	N.D				
U-soil	15	Detected	11	Detected	5	Detected
	18	Detected	13	N.D	7	N.D
	20	Detected				
	25	N.D				
Water	13	Detected	11	Detected	7	Detected
	15	Detected	13	Detected	9	N.D
	18	Detected	15	Detected		
	20	Detected	18	Detected		
	25	N.D	20	Detected		
			25	N.D		

Supplementary Table 2: Soil and water analysis results

Parameter	Soil (mg/kg)		Water (mg/L)
	D-soil	U-soil	
P	1319.39	485.28	-
K_2O	8010.45	2991.05	-
Mn	344.34	438.97	-
S	104.37	403.18	-
NaCl	77.06	128.41	-
Cl^-	-	-	6.2
pH	4.5	5.8	7.0
